# Water Corrosion of Tungsten Target for Accelerator-Driven Neutron Source

**DOI:** 10.3390/ma15103448

**Published:** 2022-05-11

**Authors:** Yupeng Xie, Qiuyu Sun, Yaocheng Hu, Xiaobo Li, Zhaopeng Qiao, Jie Wang, Sheng Wang

**Affiliations:** Shaanxi Engineering Research Center of Advanced Nuclear Energy, Shaanxi Key Laboratory of Advanced Nuclear Energy and Technology, School of Nuclear Science and Technology, School of Energy and Power Engineering, Xi’an Jiaotong University, Xi’an 710049, China; xieyupeng@stu.xjtu.edu.cn (Y.X.); qiuyu.sun@stu.xjtu.edu.cn (Q.S.); hyc1997@stu.xjtu.edu.cn (Y.H.); alexlee7@stu.xjtu.edu.cn (X.L.); qiaozhaopeng@stu.xjtu.edu.cn (Z.Q.); wangjie1@xjtu.edu.cn (J.W.)

**Keywords:** water corrosion, tungsten target, accelerator-driven neutron source

## Abstract

The water corrosion of tungsten as a target material can affect the safe operation of accelerator-driven neutron source. This paper reported the corrosion behaviors of tungsten in ultrapure water and tap water for 7, 14, 21, 30 and 60 days. Moreover, ICP-MS, XRD, XPS, SEM-EDS and LSCM were used to analyze the components in solutions, crystalline structures, chemical compositions and surface morphologies. It was found that the dissolution of tungsten, due to corrosion, reached its maximum between 30 days and 60 days in both solutions. The cube-shape substance, CaWO_4_, was the main corrosion product after tungsten in tap water. The tungsten oxide was changed from WO_3_ to WO_2_ during the corrosion of tungsten in ultrapure water. Compared with tungsten in ultrapure water, tungsten in tap water had its surface completely destroyed, with a dense diamond shape. Therefore, based on the analysis from this study, the corrosion mechanisms of tungsten in ultrapure and tap water were revealed.

## 1. Introduction

Neutron has received considerable attention in earth, energy, environmental sciences and clinical applications due to its advantages of uniqueness and non-destruction [1,2]. The types of neutron sources under construction are mainly include reactor neutron source [3], accelerator-driven neutron source [4], and isotopic neutron source [5]. Recent developments in the field of neutron sources have reignited interest in accelerator-driven neutron sources to supply high-intensity neutron beams to support and develop neutron activities [6]. Tungsten is mainly used as the target material of accelerator-driven neutron source, especially spallation neutron source [7]. For example, the China Spallation Neutron Source (CSNS) is designed to accelerate proton beam pulses to 1.6 GeV kinetic energy at 25 Hz repetition rate, striking a solid metal target (tungsten) to produce spallation neutrons. Due to the high density beam power of about 240 kW leading to high heat density—which is similar to the European Spallation Source (ESS), whereby neutrons are released from a rotating tungsten target when it is hit by 2 GeV protons provided by a superconducting linac at an unprecedented 5 MW of average beam power—the tungsten target cooling is necessary for its safe operation [8,9].

Water cooling is one of the most widely used cooling methods to decrease the target temperature. However, water corrosion will occur on the tungsten surface due to the contact of water with tungsten, thus impacting the safe operation of the target system. Li et al. reported that the cracking of tungsten occurred in a water-cooled divertor target, resulting in target failure [10]. Jiang et al. found remarkable intergranular corrosion as well as slight dissolution of the tungsten in a flowing water system at 20 °C [11]. Collins et al. demonstrated that significant weight loss occurred over a very short period during the corrosion of tungsten in water [12]. Moreover, the water corrosion of tungsten causes the decrease in the delivered neutron flux [13]. Therefore, analyzing the corrosion behavior of the tungsten target in the cooling water is essential for selecting cooling medium and anti-corrosion methods.

In this work, the corrosion behaviors of tungsten in two environments were studied to compare the corrosion effects of tap water and ultrapure water. In order to reveal the corrosion mechanism, the tungsten target was immersed in water for 7, 14, 21, 30 and 60 days, and the corrosion temperature was set to 50 °C (the standard of maximum temperature of coolant) [14]. The physicochemical characterizations as-received and corrosive tungsten samples were performed by X-ray diffraction (XRD), X-ray photoelectron spectroscopy (XPS), scanning electron microscope-energy dispersive spectroscopy (SEM-EDS) and laser scanning confocal microscope (LSCM). Additionally, the element components of residual aqueous solution were determined by using inductively coupled plasma mass spectrometry (ICP-MS). Additionally, according to the above measurements, the corrosion mechanisms of tungsten in tap water and ultrapure water were proposed.

## 2. Materials and Experiments

### 2.1. Materials

Purchased from Qinghe Haoxuan Metal Material Co., Ltd., tungsten samples in blocks with a purity of 99.95% were cut to 10 × 10 × 2 mm by laser. Before the corrosion experiments, all tungsten samples were washed with propanol and ultrapure water, and then dried at 80 °C overnight. The tap water was taken from Xi’an Jiaotong University, Xi’an, Shaanxi, China, with the major components for this study given in Table 1 from ICP-MS. Moreover, the ultrapure water used in this study was with the resistivity of 18.25 MΩ•cm.

### 2.2. Corrosion Experiments

For each corrosion experiment, 25 mL water (tap water and ultrapure water) was added in a polypropylene centrifuge tube, in which a tungsten sample was loaded. Then, the tube was kept in a temperature box at 50 °C for 7, 14, 21, 30 and 60 days. On completion of the corrosion experiments, the corrosive tungsten sample was taken out from the centrifuge tube and then washed with propanol and ultrapure water and dried at 80 °C overnight for further characterizations. Meanwhile, the residual aqueous solutions after corrosion were kept in the refrigerator for ICP-MS measurements.

### 2.3. Physicochemical Characterizations

ICP-MS measurements were performed on a spectrometer (NexIONTM 350D, Perkin Elmer, MA, USA) to determine the contents of Mg, Fe, Na and Ca, and W dissolved from the tungsten samples in residual aqueous solutions. The crystalline structures of as-received tungsten and corrosive tungsten samples were analyzed by X-ray diffraction (XRD, D8 ADVANCE, Bruker, MA, USA). The 2θ scanning range of XRD patterns was set to be 20∼60° with a step size of 0.02°. The chemical components of tungsten on the surface were detected with X-ray photoelectron spectroscopy (XPS, AXIS ULtrabld, Shimadzu, Kyoto, Japan), with an AI monochromatic energy resolution of 0.48 eV. The binding energies were calibrated with a binding energy of C 1s peak of 284.8 eV. The scanning electron microscopy with an energy dispersive spectrometer (SEM-EDS, JEOL 7800F, JEOL Ltd., Tokyo, Japan) was used to determine the morphologies and the elemental distributions on the surfaces of as-received and corrosive tungsten samples. The 3-D images for the surfaces of as-received and corrosive tungsten were collected by a laser scanning confocal microscope (LSCM, OLS4000, Olympus, Tokyo, Japan).

## 3. Results and Discussion

### 3.1. ICP-MS

**Figure 1:**
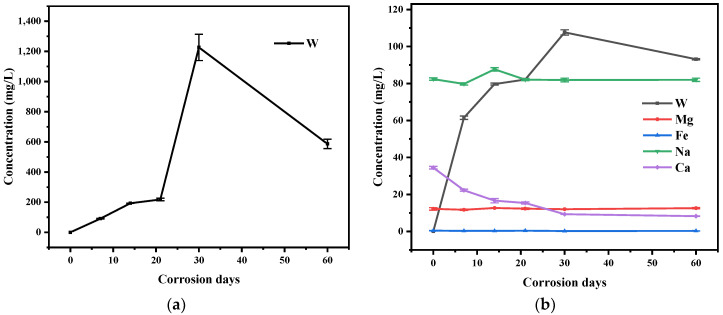
Changes in element concentration of (**a**) tungsten in ultrapure water and (**b**) tungsten in tap water for various corrosion days.

The elements concentrations of residual aqueous solutions were determined by ICP-MS (Figure 1). Since the corrosion of tungsten in ultrapure water did not involve other elements, only W changed, as shown in Figure 1a, which showed that the concentration of W increased until it reaches the maximum between 30 days and 60 days, and then decreased, as did W in tap water. This may be because the dissolved W was re-adsorbed to the surface after reaching maximum. Ca in the corrosion solution of tap water changed more obviously than other elements (Mg, Fe and Na), and the concentration decreased with the increase in corrosion time, indicating that the Ca-containing substance was the main corrosion product for tungsten in the tap water.

### 3.2. XRD

**Figure 2:**
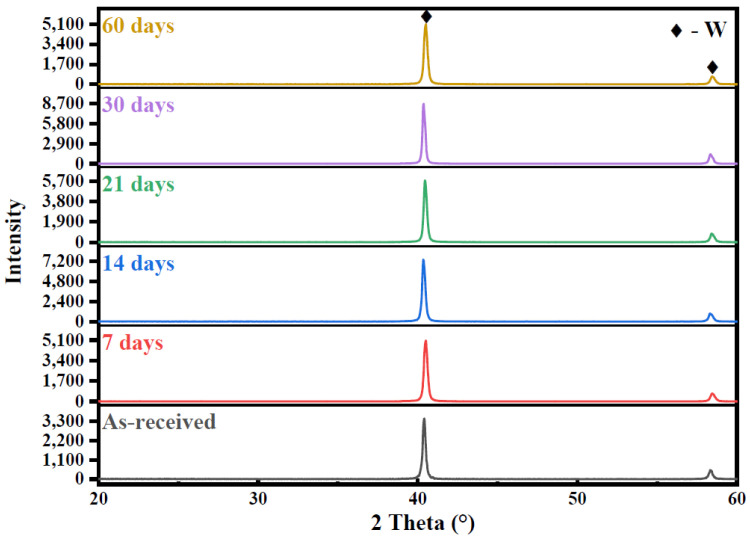
XRD patterns of as-received tungsten and corrosive tungsten in ultrapure water for 7, 14, 21, 30 and 60 days. ◆ is the sign representing a peak belong to tungsten (W).

**Figure 3:**
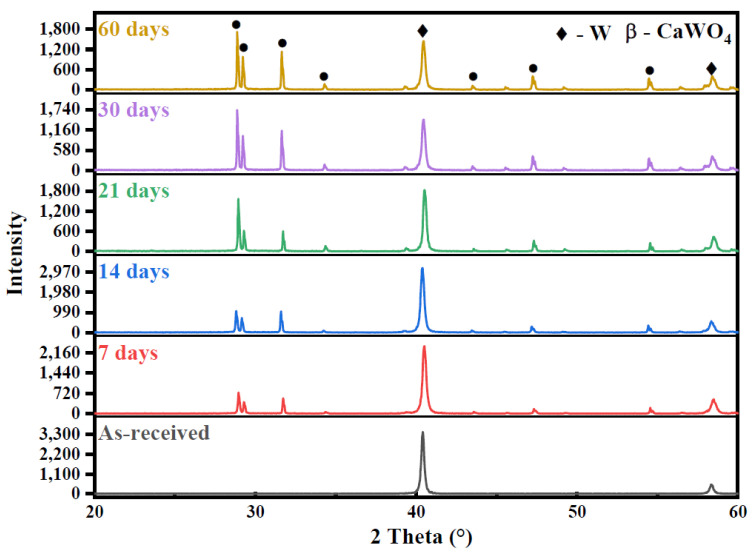
XRD patterns of as-received tungsten and corrosive tungsten in tap water for 7, 14, 21, 30 and 60 days. ◆ and • are the signs representing a peak belong to tungsten (W) and CaWO_4_, respectively.

XRD measurements were used to determine the crystalline structures of corrosion products on the tungsten surface. As shown in Figure 2, two peaks representing W at 40.43° and 58.37° were detected on the surface of as-received tungsten [15]. Moreover, no other peak was detected after corrosion, meaning that no phase change occurred on the surface during the corrosion of tungsten in ultrapure water. On the other hand, several peaks representing CaWO_4_ were observed after corrosion in tap water (Figure 3) [16]. CaWO_4_ may be the main corrosion product on the surface of tungsten in tap water. Prior study had noted that the crystallinity was related to the intensity of the strongest diffraction peak [17]. In this study, the relative intensity of the strongest CaWO_4_ peak at 28.78° gradually increased with the increase in corrosion time, indicating that the content and crystallinity of CaWO_4_ on the surface was increasing after corrosion in tap water.

### 3.3. XPS

**Figure 4:**
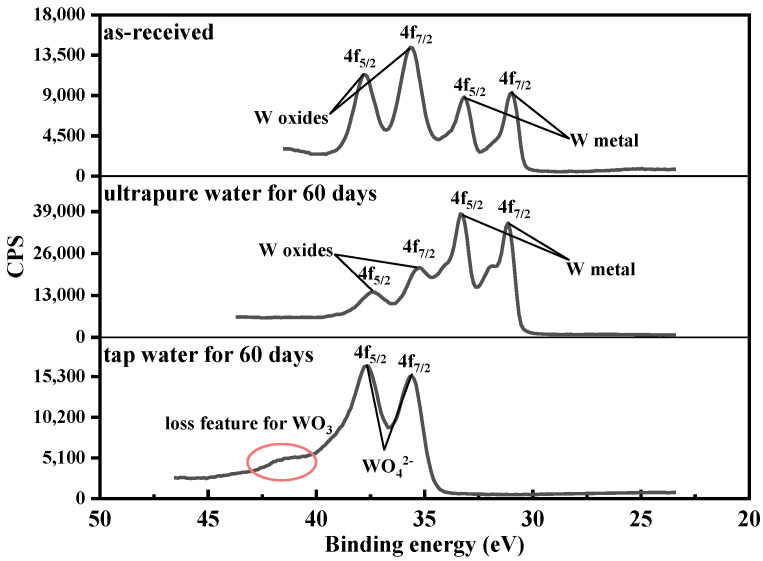
XPS spectra of the W 4f levels for as-received tungsten and tungsten in ultrapure water and tap water for 60 days.

**Figure 5:**
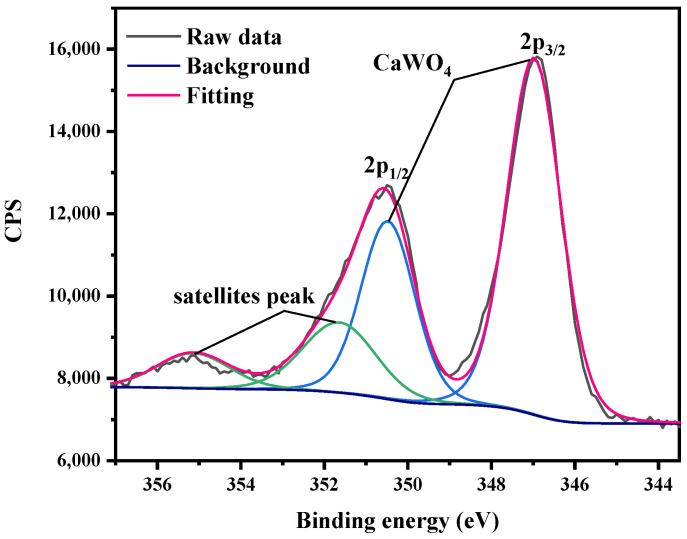
The deconvolution of spectra for the Ca 2p levels of tungsten in tap water for 60 days.

To determine the chemical compositions of tungsten surface, we characterized the as-received tungsten and the corrosive tungsten through XPS analysis. Since the tungsten in ultrapure water during the whole corrosion process had spectra with a similar shape, as did the tungsten in tap water, only spectral analysis of tungsten in tap and ultrapure water for 60 days are included here. From Figure 4, the XPS spectra of as-received tungsten and tungsten in ultrapure water included several peaks representing W oxides and W metal, respectively, and the spectra of tungsten in tap water were missing two peaks representing W metal. Moreover, a shoulder peak appeared at 41.36 eV for tungsten in tap water, which was assigned to the loss feature of WO_3_ [18]. Therefore, the peaks at 37.73 eV and 35.56 eV of tungsten in tap water were attributed to WO_4_^2−^ [19]. A pair of peaks at 350.48 eV and 346.98 eV representing CaWO_4_ and two satellite peaks at 355.14 eV and 351.64 eV were observed in Figure 5 [20], which were consistent with XRD results that the main corrosion product in tap water was CaWO_4_. WO_3_ is the most common oxide of tungsten [21]. W metal and WO_3_ can dissolve in aqueous alkaline (alkaline tap water in this study) solutions to form tungstate ions, WO_4_^2-^ (Equations (1) and (2)) [22], resulting in the disappearance of the peaks of W meal and W oxides in XPS spectra of tungsten in tap water.

(1) $${W+8\mathrm{OH}}^{-}\overset{\;}{\to }{\mathrm{WO}}_{4}^{2-}{+4H}_{2}O+6e$$

(2) $${\mathrm{WO}}_{3}{+2\mathrm{OH}}^{-}\overset{\;}{\to }{\mathrm{WO}}_{4}^{2-}{+H}_{2}O$$

**Figure 6:**
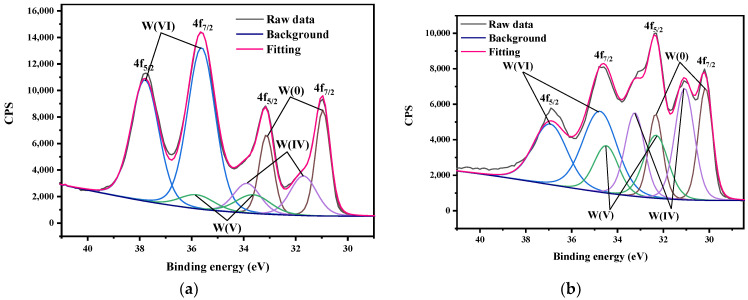
The deconvolution of spectra for the W 4f levels of (**a**) as-received tungsten and (**b**) tungsten in ultrapure water for 60 days.

To further determine the valence states of W in the surface oxides of as-received tungsten and tungsten in ultrapure water, deconvolutions of spectra on the W 4f levels were performed. As can be seen from Figure 6, the W 4f spectra consisted of several components representing different oxidation states of tungsten, and the peak positions are given in Table 2 [23]. Although these two tungsten samples had the same kind of oxide, the spectra of tungsten in ultrapure water showed a shoulder peak, indicating a possible change in the ratio between oxides. After the data analysis from XPS fitting, we found that the dominant oxidation state in as-received tungsten was W^6+^ for WO_3_ (58%), while W^4+^ (32.47%) for WO_2_ was dominant for tungsten in ultrapure water [24]. This phenomenon indicated the oxide valance change of tungsten occurred on the surface during the corrosion of tungsten in ultrapure water.

### 3.4. Surface Morphology

**Figure 7:**
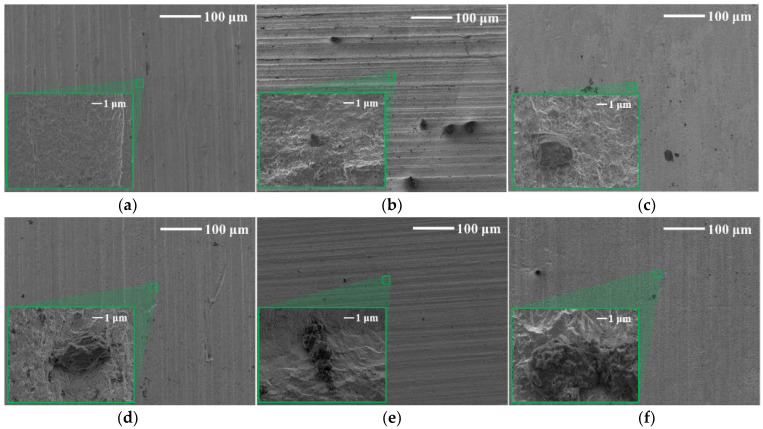
SEM images of (**a**) as-received tungsten and tungsten in ultrapure water for (**b**) 7 days, (**c**) 14 days, (**d**) 21 days, (**e**) 30 days and (**f**) 60 days. (The image in green square is with high magnification).

**Figure 8:**
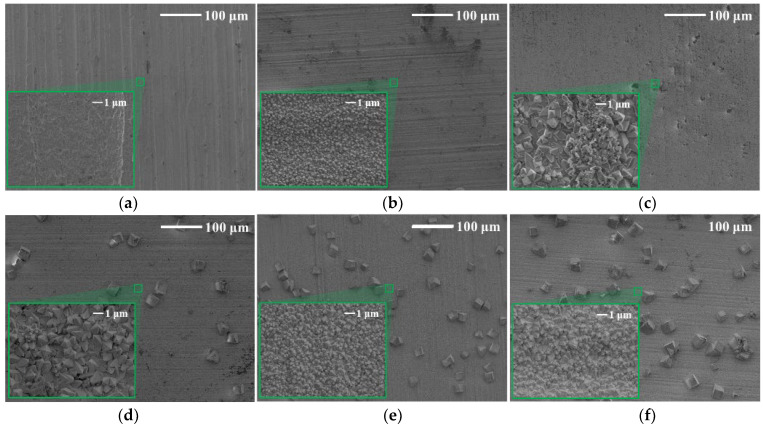
SEM images of (**a**) as-received tungsten and tungsten in tap water for (**b**) 7 days, (**c**) 14 days, (**d**) 21 days, (**e**) 30 days and (**f**) 60 days. (The image in green square is with high magnification).

The surface morphology of tungsten was observed by SEM, and the high magnification was circled with a green frame. In Figure 7, blocky corrosion products that appeared on the surface of tungsten in tap water were distributed sporadically. For tungsten corrosion in tap water (Figure 8), compared with as-received tungsten, the surface had no obvious change at low magnification before 14 days, while the surface was completely destroyed and featured a dense diamond shape at high magnification. From 21 days, cube-shaped corrosion products can be observed at low magnification and distributed more densely as the corrosion time increased.

**Figure 9:**
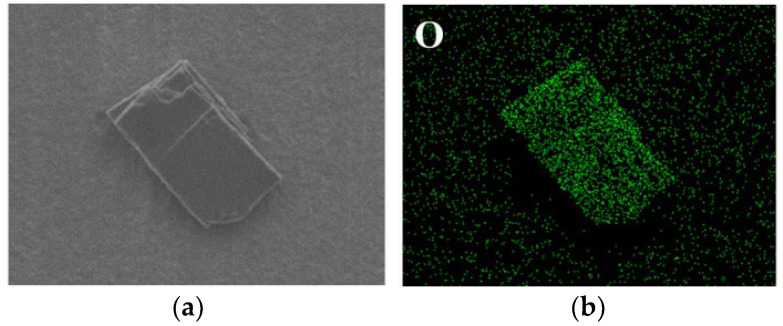
Elements distributions of the surface of tungsten in ultrapure water for 60 days. ((**a**) is the SEM image; (**b**) is the mapping of O element).

**Figure 10:**
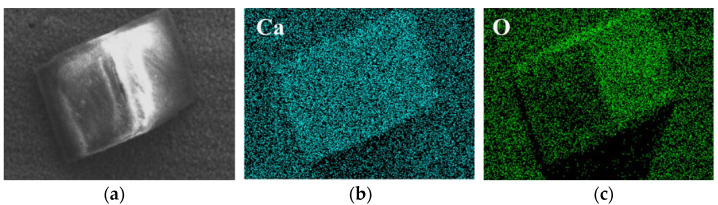
Elements distributions of the surface of tungsten in tap water for 60 days. ((**a**) is the SEM image; (**b**) is the mapping of Ca element; (**c**) is the mapping of O element).

**Figure 11:**
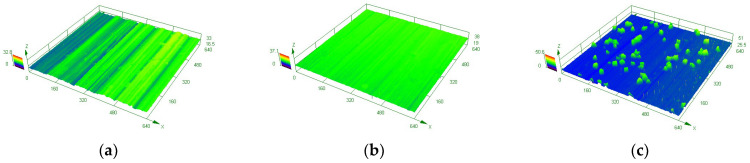
3D images of LSCM for (**a**) as-received tungsten, (**b**) tungsten in ultrapure water for 60 days and (**c**) tungsten in tap water for 60 days.

To determine the compositions of corrosion products, EDS scanning was performed on the corrosion products in both environments (Figure 9 and Figure 10). It can be found that the main component of corrosion products of tungsten in tap water was the element of O, but the current method cannot measure the specific composition. As for tungsten in the tap water, elements mapping showed the aggregation of Ca and O, which further confirmed the previous analysis that the corrosion product was calcium tungstate. Moreover, the 3D images of tungsten from LSCM demonstrated that compared with the surface of as-received tungsten and tungsten in ultrapure water, CaWO_4_ protruded significantly on the surface of tungsten after corrosion in tap water (Figure 11).

### 3.5. Corrosion Mechanism

**Figure 12:**
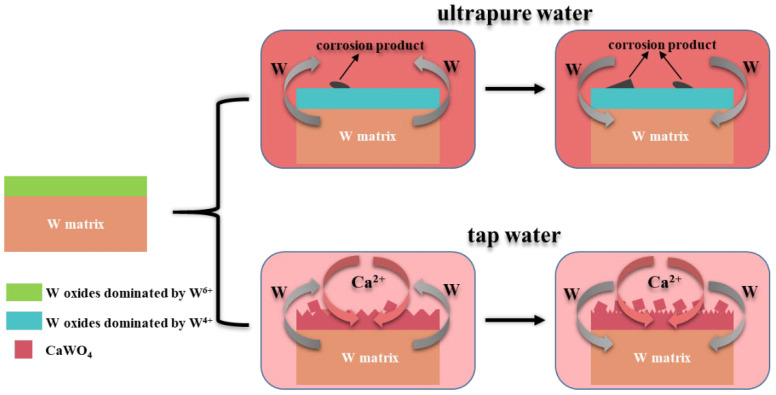
Schematic diagram of corrosion mechanism of tungsten in ultrapure water and tap water.

According to the above characterizations, the corrosion mechanisms of tungsten in ultrapure water and tap water were revealed (Figure 12). The dissolution of W reached a maximum between 30 days and 60 days in both solutions and then was re-adsorbed to the tungsten surface. In ultrapure water, with the increase in corrosion time, the dominant oxide valence on the surface changed from W^6+^ to W^4+^. After corrosion, the crystalline structures of the surface did not change, and the corrosion products were distributed in blocks. In tap water, Ca was the main element of the corrosion products. Additionally, with the increase in corrosion time, Ca gradually deposited on the tungsten surface, and combined with the WO_4_^2-^ generated on the surface of W to form CaWO_4_ on the surface in cube shape. The tungsten surface was completely destroyed due to exposure to tap water, featuring a dense diamond shape.

## 4. Conclusions

In this study, we investigated the corrosion behaviors of tungsten in ultrapure water and tap water for 7, 14, 21, 30 and 60 days. Based on the characterizations of ICP-MS, XRD, XPS, SEM-EDS and LSCM, the corresponding corrosion mechanisms were subsequently proposed, offering guidance for the selection of anti-corrosion methods. The main conclusions of this study are as follows:

(1) The corrosion-caused dissolution of tungsten reached its maximum between 30 days and 60 days in both solutions. Compared with other elements (Mg, Fe and Na), Ca in tap water was more easily adsorbed on the surface of tungsten, indicating the Ca-containing substance was the main corrosion product in tap water.
(2) CaWO_4_, the main corrosion product, was detected on the surface of tungsten after exposure to tap water. Moreover, the surface oxide valence of tungsten changed (from W^6+^ to W^4+^) during corrosion in ultrapure water.
(3) The corrosion product in ultrapure water was blocky on the tungsten surface, while CaWO_4_ was in a cube shape on the surface after corrosion in the tap water, and the surface was completely destroyed, featuring a dense diamond shape.
(4) During the construction of the neutron source, the use of ultrapure water with the resistivity of 18.25 MΩ•cm can lead to high costs. According to the results of this study, Ca^2+^ in tap water is the precursor of the main corrosion products (CaWO_4_). In order to reduce the construction cost, it is only necessary to remove Ca from tap water and use it as cooling medium.

## Figures and Tables

**Table 1:** The elements components of tap water.

	Mg	Fe	Na	Ca
Concentration, mg/L	12.3	0.404	81.9	34.5

**Table 2:** The peak positions of all W oxides in XPS spectra of as-received tungsten and tungsten in ultrapure water for 60 days.

	W(VI), eV		W(V), eV		W(IV), eV		W(0), eV	
	4f_5/2_	4f_7/2_	4f_5/2_	4f_7/2_	4f_5/2_	4f_7/2_	4f_5/2_	4f_7/2_
As-received	37.79	35.62	35.80	33.63	33.88	31.71	33.13	30.96
Ultrapure water for 60 days	37.55	35.38	35.29	33.12	34.04	31.87	33.28	31.11

## Data Availability

Data available on request from the authors.
